# Anti-Stress Action of an Orally-Given Combination of Resveratrol, β-Glucan, and Vitamin C

**DOI:** 10.3390/molecules190913724

**Published:** 2014-09-03

**Authors:** Vaclav Vetvicka, Jana Vetvickova

**Affiliations:** Department of Pathology, University of Louisville, Louisville, KY 40202, USA

**Keywords:** resveratrol, β-glucan, vitamin C, stress, immunomodulator, cytokines

## Abstract

Stress has repeatedly been found to reduce the abilities of the immune system to fight against individual attacks. The current dissatisfaction with classical medications has led to more attention being focused on natural molecules. As recent studies have suggested that some bioactive molecules can have synergistic effects in stimulation of immune system and reduction of stress, we have evaluated the stress-reducing effects of the resveratrol-β-glucan-vitamin C combination. We found that compared to its individual components, this combination was the strongest reducer of stress-related symptoms, including corticosterone levels and IL-6, IL-12 and IFN-γ production.

## 1. Introduction

β-Glucans are structurally complex homopolymers of glucose, isolated from various sources including yeast, fungi, and wheat. Their role as biologically active immunomodulators has been well documented for more than 50 years (for reviews see [1,2,3,4]). The positive effects of β-glucan treatment have been repeatedly confirmed in clinical trials [5]. Despite the extensive research, the links between physicochemical properties such as molecular weight or 1,4 *vs.* 1,6 branching are not fully established. It should be noted that barley and oat contain mostly 1,3/1,4 linked glucans, whereas mushrooms and yeasts contain mostly but not only1,3/1,6 linked glucans. However, there is no clear-cut evidence of particular effects being linked with only 1,4 or 1,6 glucans, similarly as it is not linked with glucan from a particular source.

Studies evaluating the effects of β-glucan on stress are rather rare. Mushroom-derived β-glucan was found to reduce restrain stress in mice [6], and oat β-glucan managed to lower the effects of exercise stress [7]. Positive findings were also found in highly-stressed subjects [8]. When several different types of β-glucans were directly compared as anti-stress molecules, the level of activity was found to widely differ among individual β-glucans [9].

β-Glucan is clearly not the only known immunomodulator. More and more companies and/or scientists are experimenting with improvements of β-glucan action by adding additional immunomodulators. Therefore, combinations of various natural immunomodulating agents are becoming more popular. The most common feature of these mixtures is β-1,3-glucan, which was found to have strong synergy with vitamin C almost 20 years ago [10], probably due to the fact that vitamin C stimulates the same types of immune responses as β-glucan. Later studies found strong synergy between yeast-derived β-glucan and humic acid, with potentiated phagocytosis, cytokine release, and protection against hepatotoxicity [11,12]. Recently, our group found that yeast β-glucan combined with resveratrol and vitamin C showed significant improvements in stimulation of both cellular and humoral immunity, including anticancer activities [13]. Our findings indicated that adding *Withania somnifera* extract to the maitake mushroom β-glucan significantly regulates stress-induced increase on corticosterone levels [14] and were further supported by a study showing stronger anti-stress effects of yeast β-glucan and vitamin C [9,15], that led us to the current study evaluating anti-stress activities of a β-glucan-resveratrol-vitamin C mixture.

## 2. Results and Discussion

### 2.1. Results

**Figure 1:**
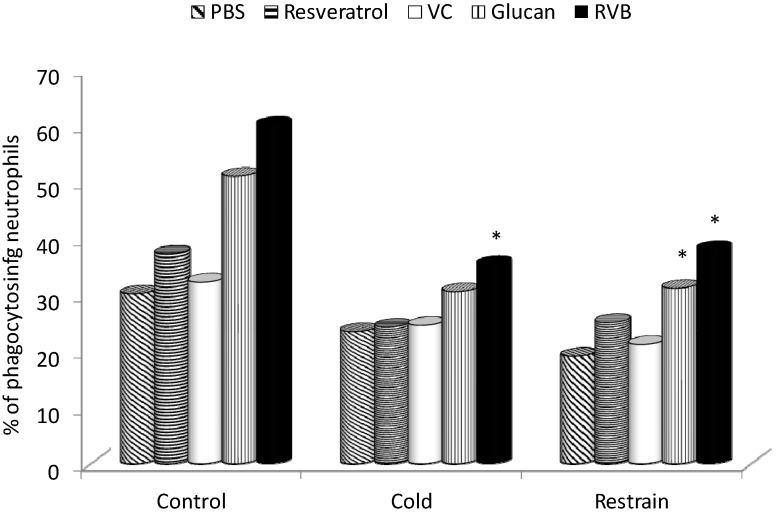
Effect of an oral administration of different samples on phagocytosis by peripheral blood granulocytes in stressed mice. * Represents significant differences between stressed-control (PBS) and samples at *p* ≤0.05 level. PBS—phosphate buffered saline; VC—vitamin C; RVB—resveratrol-vitamin C-glucan combination. Ten mice/group.

The rationale for a combination of β-glucan with two other bioactive molecules was based not only on their commercial availability and established effects, but most importantly on the previously described synergistic action. β-Glucans are generally considered to be strong stimulators of the cellular branch of immune reactions, with macrophages and granulocytes being the most important targets. Therefore, our first experiments were focused on the role of β-glucan in stress-related changes in phagocytic activity. We used synthetic polymeric microspheres (HEMA), since their use, dose and timing are already well established in β-glucan studies [16]. Results summarized in Figure 1 show that both types of stress caused a significant (up to 30%) reduction of phagocytic activity. β-Glucan and the resveratrol-vitamin C-glucan (RVB) combination were able to return the phagocytosis to normal levels. However, this stimulation never reached the levels of β-glucan or RVB activation in control animals.

**Figure 2:**
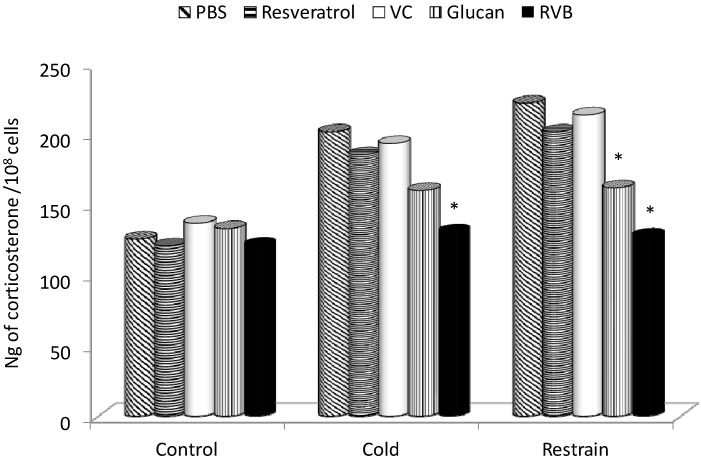
Effect of 14 days of feed supplementation on stress-induced levels of corticosterone. * Represents significant differences between stressed-control (PBS) and β-glucan samples at *p* ≤0.05 level. PBS—phosphate buffered saline; VC—vitamin C; RVB—resveratrol-vitamin C-glucan combination. Ten mice/group.

The following experiments measured the level of corticosterone in serum. In the control group, feeding with either of the individual components of the mixture did not alter the level of corticosterone. On the other hand, both types of stress resulted in a significant increase of corticosterone. Resveratrol alone or vitamin C alone offered no protection, while β-glucan-supplementation blocked the increase to some extent. However, the combination offered complete inhibition of the stress-related increase of corticosterone (Figure 2).

**Figure 3:**
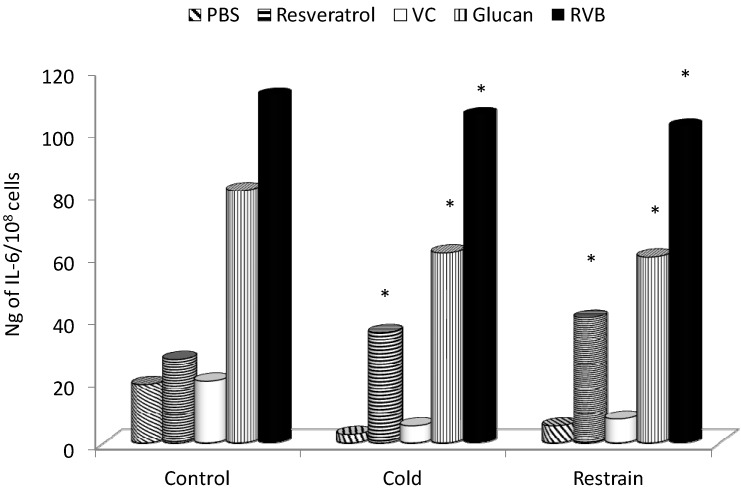
Effect of 14 days of feed supplementation on stress-induced levels of IL-6. * Represents significant differences between stressed-control (PBS) and tested samples at *p* ≤0.05 level. PBS—phosphate buffered saline; VC—vitamin C; RVB—resveratrol-vitamin C-glucan combination. Ten mice/group.

The basal production of IL-6 by Con A-stimulated splenocytes was low (app. 20 ng) and was significantly increased only by β-glucans and RVB combination. Both types of stress reduced the IL-6 secretion, but only resveratrol, β-glucan and RVB combination increased IL-6 secretion above control (PBS) levels. In all cases, the combination elicited the highest secretion of IL-6, which was comparable to control values (Figure 3).

**Figure 4:**
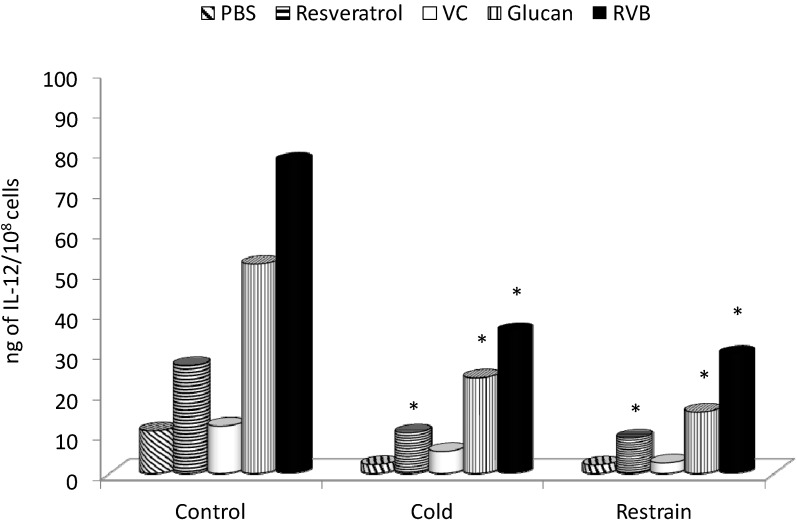
Effect of 14 days of feeding with individual samples or combinations on stress-induced levels of IL-12. * Represents significant differences between stressed-control (PBS) and tested samples at *p* ≤0.05 level. PBS—phosphate buffered saline; VC—vitamin C; RVB—resveratrol-vitamin C-glucan combination. Ten mice/group.

Similar results were found when we measured IL-12 (Figure 4). Both types of stress caused significant inhibition of IL-12 formation, which was strongly reduced by resveratrol, β-glucan or RVB combination.

**Figure 5:**
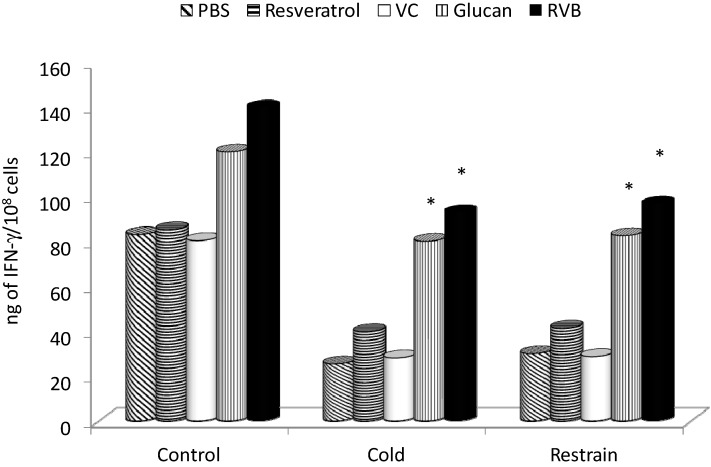
Effect of 14 days of feeding with individual samples of combinations on stress-induced levels of IFN-γ. * Represents significant differences between stressed-control (PBS) and tested samples at *p* ≤0.05 level. PBS—phosphate buffered saline; VC—vitamin C; RVB—resveratrol-vitamin C-glucan combination. Ten mice/group.

In the case of IFN-γ, both β-glucan and the RVB combination stimulated the secretion in control mice. Both types of stress caused over 60% reduction in IFN-γ secretion, and this reduction was improved by resveratrol (significant improvement in cold-mediated stress only). β-Glucan alone managed to boost the secretion to almost a normal level in stress-inhibited animals (Figure 5). However, the effects of β-glucan alone or RVB combination were again much stronger.

Both types of stress managed to significantly increase both systolic and diastolic blood pressure. Supplementation with RVB significantly improved both levels (Table 1). Neither stress nor RVB addition changed the heart rate. Vitamin C, β-glucan or resveratrol alone did not change heart rate values (data not shown).

**Table 1:** Effects of tested material on blood pressure and heart rate.

	RVB	SBP	DBP	Heart Rate
No stress	-	122 ± 2	102 ± 2	585 ± 9
No stress	+	120 ± 3	101 ± 2	572 ± 8
Cold	-	141 ± 4	110 ± 3	653 ± 9
Cold	+	130 ± 2 *	103 ± 2 *	633 ± 9
Restrain	-	147 ± 3	117 ± 4	667 ± 9
Restrain	+	126 ± 4 *	105 ± 2 *	653 ± 7

SBP—systolic blood pressure (mmHg); DBP—diastolic blood pressure (mmHg); Data represent mean ± SD. * Significant differences between control sample (PBS) and sample with RVB. Ten animals in each group measured at the endpoint of the study.

### 2.2. Discussion

Individual natural molecules or complex materials have been sought for centuries. The current low satisfaction with classical drugs has focused more attention on natural products. β-Glucans, as probably the most studied natural immunomodulators, offer such a possibility. Chemically, β-glucan is a polymer of glucose. There are various natural sources of β-glucans; however, they are most frequently prepared from yeast and fungal cell walls (for more details, see the recent monograph [17]). Despite more than 10,000 scientific reports on β-glucan’s properties, some recent studies have suggested that the addition of one or more biologically active molecules can improve bioactivity of β-glucan. The first and most studied molecule with such synergistic effects is vitamin C [10,18,19]. Similarly, β-glucan’s effect can be potentiated by addition of humic acid [11,12] or *Ashwagandha* extract [14].

Our study was based on two preliminary findings. First, recent studies suggested that β-glucan can restore some immune functions depressed by stress [6,9,20]. Second, the β-glucan-resveratrol-vitamin C combination has superior immunostimulating properties, including anti-cancer effects [13,21].

The connection between stress and immune reactions is well-established, with the hypothalamic-pituitary-adrenal axis [22] and particularly corticosterone playing the major role [23]. As β-glucans are often considered to be primarily the stimulators of nonspecific immunity, we first evaluated the effects on phagocytic activity. Using a model of synthetic hydroxyethylmethacrylate particles, we found that both types of stress significantly decreased the phagocytic activity of peripheral blood neutrophils. In both cases, the feeding with the RVB combination increased the phagocytic activity to the level of control (PBS) phagocytosis. However, the phagocytosis never reached levels obtained by either β-glucan or RVB in control mice.

In addition to the direct effect on various immunocytes, the biological effects of β-glucans are caused by potentiation of a secretion of several cytokines such as TNF-α, IFN-γ, IL-1 and IL-2. This cytokine stimulating activity is dependent on numerous factors including β-glucan’s origin, molecular weight, and the triple helix conformation [1]. Similarly, resveratrol was found to stimulate production of IL-2 and IL-4 [24] and TNF-α [25]. In our study we used three different cytokines, secretion of which was strongly inhibited by the stress. IL-12 is primarily secreted by cells such as macrophages and dendritic cells as a response to microbial factors challenge. It is also considered to be a T-cell stimulating factor [26]. IL-6 is a pleiotropic cytokine produced by a variety of cells. It is not only involved in inflammation and infection responses but also in the regulation of metabolic, regenerative, and neural processes. IL-6 is secreted by T cells and macrophages to stimulate immune response, e.g., during infection and after trauma (for review see [27]). IFN-γ is a cytokine critical for both innate and adaptive immunity (for a review see [28]). 

In our study, we showed that both types of stress strongly inhibited the production and/or release of all three tested cytokines, and supplementation with RVB combination reversed this suppression. β-glucan alone and resveratrol alone also offered strong protection against stress-related inhibition, but the effects were not as strong as the effects of combination. Kimura *et al.* found similar β-glucan-mediated restoration of stress-related inhibition of cytokine production [6].

Significant increase of corticosterone levels after both types of stress are in agreement with previous studies (for review see [29]). β-Glucan alone and RVB combination both reduced the stress-related boost of corticosterone production, which was hypothesized to help regulate the negative effects of stress. These effects were already described for β-glucan [9], but the synergistic effects of the β-glucan-resveratrol-vitamin C combination were surprising.

The effects of stress on blood pressure are well documented [30]. Therefore, our findings of stress-induced changes of blood pressure are not surprising. Only the combination helps to reduce these effects, individual components were without effects. Our data were rather surprising, as in mouse models, the hypertension is usually more connected with obesity, salty diet or genetic disposition [31].

Our current study demonstrates that the already established synergistic effects of β-glucan and resveratrol [32,33] can be further improved by adding vitamin C. These molecules, particularly when sufficiently purified and well characterized, have pleiotropic effects reaching beyond the originally suggested modulation of nonspecific immunity. It seems possible to develop this combination into a stress-reducing supplement.

## 3. Experimental Section

### 3.1. Animals

Female, 8-week-old BALB/c mice were purchased from the Jackson Laboratory (Bar Harbor, ME, USA). All animal work was done according to the University of Louisville IACUC protocol. Animals were sacrificed by CO_2_ asphyxiation. At least 10 animals were used in each group.

### 3.2. Material

Yeast-derived insoluble β-glucan #300 (over 86% pure) were purchased from Transfer Point (Columbia, SC, USA), vitamin C from Sigma (St. Louis, MO, USA), and resveratrol from Suan Farma (Paramus, NJ, USA). Based on HPLC analysis, it is 98.2% pure *trans*-resveratrol isolated from *Polygonum cuspidatum*. All these substances were administered orally (gavage) at doses of 100 μg each.

### 3.3. Phagocytosis

The technique employing phagocytosis of synthetic polymeric microspheres was described earlier [15]. Briefly, peripheral blood cells were incubated with 0.05 mL of 2-hydroxyethyl methacrylate particles (HEMA; 5 × 10^8^/mL). The test tubes were incubated at 37 °C for 60 min, with intermittent shaking. Smears were stained with Wright stain. The cells with three or more HEMA particles were considered positive. The same smears were also used for evaluation of cell types.

### 3.4. Restrain Stress Protocol

Mice were subjected to restraint stress according to a method described earlier [6]. Briefly, mice were restrained for 6 h for 5 days (starting at day 9) in a 50 mL conical polypropylene centrifuge tubes in which holes had been drilled. Since the restrained mice could not access food and water, the control group of mice was similarly deprived food and water. Individual samples were administered once daily orally for 14 days, starting at day 0.

### 3.5. Cold Stress Protocol

Mice were subjcted to cold stress [34] by incubation for 60 min at 4 °C for two weeks. For the same time interval mice received individual samples administered orally by gavage. The feeding was done once per day at the same hour.

### 3.6. Measurement of Serum Corticosterone

On day 14, blod samples were collected by venipucture from mice under pentobarbital anesthesia. Obtained sera were collected and stored at −80 °C before assay. Serum corticosterone was measured using an ELISA kit (Dignostics Systems Lab, Webster, TX, USA) according to the manufacturer’s instructions.

### 3.7. Cytokine Production

Purified speen cells (2 × 10^6^/mL in RPMI 1640 medium with 5% FCS) were added into wells of a 24-well tissue culture plate. After addition of 1 μg of concanavalin A, cells were incubated for 48 h. in a humidified incubator (37 °C, 5% CO_2_). At the endpoint of incubation, supernatants were collected, filtered through 0.45 μm filters and tested for the presence of IL-6, IL-12 and IFN-γ. Levels of individual cytokines were measured using a Quantikine mouse IL-6, IL-12 or IFN-γ kit (R&D Systems, Minneapolis, MN, USA).

### 3.8. Blood Pressure

Blood pressure nd heart rate were monitored using the CODA Monitor (Kent Scientific, Torrington, CT, USA) according to the manufacturer’s recommendation.

### 3.9. Statistics

Student’s *t*-test wa used to statistically analyze the data.

## 4. Conclusions

In recent years, the combined use of natural immunomodulators is getting more attention. In our study, we focused on three modulators—well established resveratrol, β-glucan and vitamin C. We found that compared to the individual components, this combination was the strongest reducer of stress-related symptoms including corticosterone levels and IL-6, IL-12 and IFN-γ production.
